# Seasonality of Fire Weather Strongly Influences Fire Regimes in South Florida Savanna-Grassland Landscapes

**DOI:** 10.1371/journal.pone.0116952

**Published:** 2015-01-09

**Authors:** William J. Platt, Steve L. Orzell, Matthew G. Slocum

**Affiliations:** 1 Department of Biological Sciences, Louisiana State University, Baton Rouge, Louisiana, United States of America; 2 Avon Park Air Force Range, Avon Park, Florida, United States of America; DOE Pacific Northwest National Laboratory, UNITED STATES

## Abstract

Fire seasonality, an important characteristic of fire regimes, commonly is delineated using seasons based on single weather variables (rainfall or temperature). We used nonparametric cluster analyses of a 17-year (1993–2009) data set of weather variables that influence likelihoods and spread of fires (relative humidity, air temperature, solar radiation, wind speed, soil moisture) to explore seasonality of fire in pine savanna-grassland landscapes at the Avon Park Air Force Range in southern Florida. A four-variable, three-season model explained more variation within fire weather variables than models with more seasons. The three-season model also delineated intra-annual timing of fire more accurately than a conventional rainfall-based two-season model. Two seasons coincided roughly with dry and wet seasons based on rainfall. The third season, which we labeled the fire season, occurred between dry and wet seasons and was characterized by fire-promoting conditions present annually: drought, intense solar radiation, low humidity, and warm air temperatures. Fine fuels consisting of variable combinations of pyrogenic pine needles, abundant C4 grasses, and flammable shrubs, coupled with low soil moisture, and lightning ignitions early in the fire season facilitate natural landscape-scale wildfires that burn uplands and across wetlands. We related our three season model to fires with different ignition sources (lightning, military missions, and prescribed fires) over a 13-year period with fire records (1997–2009). Largest wildfires originate from lightning and military ignitions that occur within the early fire season substantially prior to the peak of lightning strikes in the wet season. Prescribed ignitions, in contrast, largely occur outside the fire season. Our delineation of a pronounced fire season provides insight into the extent to which different human-derived fire regimes mimic lightning fire regimes. Delineation of a fire season associated with timing of natural lightning ignitions should be useful as a basis for ecological fire management of humid savanna-grassland landscapes worldwide.

## Introduction

Fire is prominent in terrestrial ecosystems, particularly savanna and grassland biomes. Studies of changing climates and vegetation indicate global ascendancy of such fire-dominated ecosystems during the latter part of the Cenozoic [1]. Seasonal climates with pronounced wet/dry seasons and associated thunderstorms that produce lightning provide predictable ignitions of wildfires that can spread across landscapes in flammable vegetation [2–5]. Evolutionarily-derived fire-vegetation feedbacks [6, 7] in a climatic context result in pyrogenic ecosystems, especially savannas and grasslands that are dominated by flammable C4 grasses and contain trees and other ground layer plants that produce pyrogenic fuels [8]. Evolutionary relationships involving vegetation-fire feedbacks in these fire-frequented landscapes [9–12] may reduce inherent variation in fire regimes that otherwise would be exogenously controlled.

Humans now exert control over fire regimes worldwide. Such control typically involves changes, intentionally or unintentionally, in characteristics of fire regimes [5, 13–16]. Adaptation for aspects of historical fire regimes often is greatly reduced in importance in human-engineered fire regimes, changing ecological relationships among plant species and resulting in threats to species and even entire ecosystems adapted for historical rather than current fire regimes. In addition, the scientific focus on fire as a disturbance interrupting ongoing changes in vegetation composition rather than an environmental selection pressure has resulted in evolutionary fire-vegetation relationships being underappreciated [9, 17, 18]. Only recently has the idea emerged that conservation of entire fire-adapted floras and ecosystems depends on understanding historical fire regimes and on managing human fire regimes so that they mimic historical fire regimes [19, 20]. Such a shift in concepts related to fire has resulted in a need to study components of fire regimes worldwide [21].

Seasonality is a key component of fire regimes crucial to understanding ecological and evolutionary roles of vegetation-fire relationships. Natural fire regimes are inherently messy in nature; variation occurs in climatic conditions involved in ignition and spread of fires in landscapes that are themselves variable. In addition, patterns of natural fire seasonality now are blurred by anthropogenic ignitions [5]. Burning by humans outside the period of natural lightning ignitions has altered fire seasons [9], often by preempting or replacing lightning ignitions in fire-prone ecosystems [16, 22, 23]. As a result, the concept of fire seasonality often has been expanded to include all times when conditions are favorable for fires (both natural and anthropogenic ignitions) or all times when fires are ignited, regardless of source, within the year [24–26]. The consequence is that seasonality remains undervalued as a primary aspect of natural fire regimes [27, 28], despite its ecological and evolutionary significance, especially in frequently burned savannas and grasslands [9, 29, 30].

Concepts of natural fire seasonality emerge from empirical field studies of fire regimes. Fire seasons are most often delineated as a period of time that fires typically occur or that they are likely to be of high intensity or burn large areas of land. Characterizations tend to be ad hoc in nature, only loosely related to climatic conditions at the times of ignition and fire spread [27]. For example, in subtropical Florida a wildfire season occurs during what is often called the “transition” between the dry and wet seasons, when fine fuels are dry at the advent of convective sea-breeze storms that produce lightning strikes that ignite those fuels [31–33]. A similar process in northern Australia is termed the “build-up” [4, 34]. In the southwestern United States, an arid “foresummer” intervenes between a cool wet season (winter/spring) and summer monsoonal rains [35, 36]. In regions with a Mediterranean climate, warm months with strong winds (e.g., Santa Ana winds in California or foehn winds in Europe) are considered a fire season [37, 38]. The underlying basis of what appear to be fire seasons has not been explored.

Climate-fire related indices have attempted to describe fire seasonality. One approach has used remotely sensed data to indicate fire frequency or area burned. Global maps of fires over time [39] have been used to examine intra-annual variation in fires [24, 27, 40]. Using such remotely sensed proxies to predict seasonality of fire may underestimate peak fire months, lengthen the fire season, and emphasize human-caused temporal shifts in the timing of the fire season [41]. In the southeastern United States one study using satellite-based measurements of area burned targeted February as the peak fire month within a fire season spanning January to March [42], but a similar study identified a 10 month fire season [43]. A second approach has been to use single variables known to influence fire. For example, temporal occurrences of lightning have been used [44–46], which can lead to erroneous assumptions about timing of actual lightning ignitions and area burned. A third approach has been to use a fire weather (or danger) index to determine the likelihood of wildfires. In Florida fire season severity indices [47] showed that 65% of area burned was from January to June. Such indices have attempted to represent much of the complexity of fires by combining estimates of fuel availability (dryness) with weather variables known to influence fire (generally wind speed, relative humidity, and temperature) [21]. A fourth approach has integrated remotely sensed fire spatial data, fire- vegetation models (Dynamic Global Vegetation Model), and fire metrics (fuels, productivity, seasonal rainfall, area burned, etc.) into a meta-analysis to describe fire seasonality [27, 48, 49].

Climate indices have advanced our understanding of fire seasonality, but they also tend to be removed from direct causal relationships. In addition, relying on single metrics to delimit fire seasons discounts much of the underlying complexity governing fire behavior. For example, models that relate precipitation to area burned might predict increases in area burned with increases in precipitation because production of fine fuels is enhanced, but predictions also might indicate reductions in area burned because the dry season is reduced and so fine fuels are less dry at the times of fires [3, 50]. More direct approaches are needed to investigate relationships between climatic conditions (i.e., weather variables) and fires ignited naturally by lightning and accidentally/purposefully by humans. Thus, ecological effects of different ignition sources that change fire seasonality could be investigated and used to evaluate the extent to which humans are modifying fire regimes in ways that threaten natural ecosystems. Such approaches should provide more effective direction for restoration and management of fire regimes that incorporate important evolutionary components, especially in those regions with a long history of fire.

We addressed fire seasonality in one region of the North American Coastal Plain dominated by subtropical, seasonal savannas and grasslands. We quantitatively characterized intra-annual variation in a suite of weather variables known to affect fire spread at the Avon Park Air Force Range in south-central Florida. We used cluster analysis of weather variables known to influence fire behavior in southern Florida to develop models that grouped days into seasons. We reasoned that our approach should reveal conventional wet and dry seasons. Because our analysis focused specifically on variables known to influence fire spread [51], we hypothesized that it also should reveal clusters representing fire seasons. Our analyses suggested a three-season model with an annual fire season that was highly associated with local weather conditions that facilitate fires and that occurred annually between the dry and subsequent wet season. Our three-season model provided a better fit than the conventional rainfall model with dry and wet seasons [2].

We used our three-season fire weather model to analyze fire data at the Avon Park Air Force Range. We analyzed seasonal timing and area burned during a 13-year period based on ignition source: lightning, military missions, and prescribed fires. We specifically asked: (1) Does defining fire seasons using fire weather variables result in different seasonal patterns for fires with different ignition sources? (2) Do peak fire modes at specific times of the year that emerge from analyses of fire weather variables correspond to peak timing and largest area burned in lightning or human ignitions? (3) To what extent does fire resulting from human ignitions mimic or deviate from fires ignited by lightning? Our study provides insight into the ecological and evolutionary implications of fire seasonality in a region recognized as a biodiversity hotspot and in which the endemism is highly associated with pyrogenic savannas and grasslands. It further provides a scientific basis for constructing and adjusting fire regimes produced by management programs based on the similarities to natural fire regimes.

## Methods

### Study site

Our study focused on the Avon Park Air Force Range (hereafter, APAFR) in Polk and Highlands counties in south-central Florida (27°35’ N, 81°16’ W). The 42,430 ha military installation, located in the interior of the Florida peninsula, north of Lake Okeechobee and within the Everglades headwater region, was established during World War II for practicing air-to-ground missions. APAFR contains 38,000 ha of natural vegetation that is subject to recurrent fire. Of this acreage, 23,000 ha comprise diverse fire-maintained savanna-grassland landscapes [52]. These pine savannas and grasslands are centered within what historically encompassed over 643,600 ha of savanna-grasslands in central peninsular Florida [53, 54], and the more than 116,510 ha of grasslands in the southern peninsula comprise the largest subtropical grasslands in the United States. Currently, the fire regime is governed by a complex set of variables, including a seasonal climate, three ignition sources (lightning, military, and prescription), a fire-filtered flora, and plant communities that vary in hydroperiod, flammability, and fire history.

The seasonal humid subtropical climate of southern Florida has been characterized as having annual dry and wet seasons [55]. We previously defined these two seasons [2] for the region using cumulative rainfall anomalies [56]. Our analyses, based on data over a 58 year period (1950–2007), revealed that the wet season lasts on average 134 days (May 21 to October 1), and generates 89 ± 27 cm yr^-1^ (mean ± 1 SD) rainfall. In contrast, the much longer dry season lasts on average 231 days (October 2 to May 20), but generates about half the rainfall (42 ± 15; mean ± 1 SD cm yr^-1^). In addition, onset dates and durations of the wet and dry seasons are variable, with standard deviations of about one month for onset dates and greater than a month for durations [2]. Some of this variation is associated with ENSO oscillations [3].

Peninsular Florida is a global hotspot for lightning. Strike densities on the order of 10–15 cloud-ground strikes per square kilometer annually [57] historically resulted in fire return intervals that averaged around two years in the region prior to twentieth century alterations of the landscapes by humans (J.M. Huffman, W.J. Platt, and S.L. Orzell, unpublished data). Currently, anthropogenic fires are now the most common ignition source at the APAFR and elsewhere in the region [58, 59], and are often set earlier in the year, thereby decreasing the frequency and size of lightning fires. For example, according to the APAFR’s fire records (1997–2009), lightning fires accounted for (4%) total area burned, while military ignitions (caused by bombs, flares, rockets, and some ordnance) accounted for three times as much, with the remainder resulting from prescribed fires with human ignitions. From the early 1940s through the 1980s, during widespread fire suppression in central Florida [60–62], APAFR experienced an uninterrupted history of burning from lightning and military ignitions. Although wildfires have been influenced by landscape fragmentation (disked firebreaks, roads, etc.) and suppression efforts, these often have not been sufficient to control wildfires during extreme weather conditions. Prescribed fires at APAFR have been ignited by fire management personnel since the 1970s on a three-year burn rotation, a standard practice throughout much of the southeastern United States for managing pinelands and reducing fire danger [45, 63, 64].

Natural fires are not random. Studies in the region [44, 65] and elsewhere [66–68] show that lightning fires burn the largest areas within a narrow window prior to the mid wet season peak of lightning strikes during the convectively active warm, wet season (May thru September) in southern Florida [44]. For example, at the APAFR 74% of the recorded area burned by lightning fires occurred from 21 days before onset of the wet season to 7 days afterwards (APAFR fire records, 1997–2009). Similar trends have been noted in Everglades National Park (~200 km south of the APAFR) [44] and the complex of federally owned lands at Cape Canaveral, 125 km to the northeast [65]. Dendrochronological work in Florida, including APAFR, suggests similar seasonal timing of lightning fires back at least to the 16^th^–17^th^ centuries ([69, 70], J.M. Huffman, W.J. Platt, and S.L. Orzell, unpublished data).

Natural fires result from three synergistic conditions: fuel growth, availability and ignition timing [44, 65, 67]. Fuels accrue during the wet season, when plentiful rainfall and warm temperatures allow rapid growth of graminoids [71–73]. Fuels gradually become desiccated and cured during the subsequent dry season, allowing for fuel connectivity across the landscape in the late dry season. This is pronounced during La Niña-induced droughts [2, 3, 33], which desiccate even the lowest elevation wetlands. Lightning then ignites well-connected biomass fuels during dry conditions prior to the onset of the wet season, allowing for landscape-level wildfires [2, 34, 51]. Large lightning wildfires thus occur earlier than the mid-wet season peak of lightning strikes in Florida, when there is decreased likelihood of fire spread resulting from increased fuel moisture, precipitation, and inundation of wetlands.

### Data analysis

In this study, we used five weather variables (relative humidity, solar radiation, air temperature, wind speed, and soil moisture) that influence wildfire size at APAFR. We thus augmented existing data from our prior work [51] and added new components to conduct our study. No other area within the region has the level of data on weather that permit the analyses we conducted. Mean daily values for the first four variables were obtained from a climate station (S65CW) located 20 km southeast of the installation (data obtained using the DBHYDRO browser of the South Florida Water Management District; http://www.sfwmd.gov/dbhydro). For soil moisture, values at 30–60 cm soil depth (measured in mm) were obtained from the Experimental Surface Water Monitor (ESWM), a daily analysis of hydrologic conditions throughout the continental United States (www.hydro.washington.edu/forecast/monitor; [74]. This project has a 0.5° resolution; we used soil moisture data from a grid point (27.75° N, 81.25° W) located 10 km north of the APAFR. We used data collected over a 17-year period, 1993–2009 (6,205 days). We refer to these variables as “fire-weather variables.” Although soil moisture is not technically weather, it is a primary determinant of moisture and humidity at ground level. It thus influences moisture content of fine fuels and litter at the ground surface, and hence likelihoods of fire spread. As such, we consider it an important fire-weather variable.

The data set potentially contains many associations among the weather variables. Some are seasonally constrained, while others span seasons (e.g., storm fronts) or occur only during particular years (e.g., phases of the El Niño-Southern Oscillation). Any approach to identify fire seasons logically might first attempt to detect these associations and determine which appear the most interesting or useful. Caution is needed, however, to avoid over-fit patterns generated by random error. Based on these concerns, we went through a three-step approach to: (1) generate models containing potential associations, (2) sort through the models to find those that appeared the most useful and generated patterns attributable to seasons, and (3) ensure that candidate models were not over fit. The engine of our approach was cluster analysis, and our stepwise procedure mirrors those used in similar studies [75]. We then compared the models that were selected using the above three-step criterion. We selected what we called our “most-representative” model and conducted two additional steps to examine how effectively that model characterized fire weather and seasonal patterns of fires with different ignition sources. Lastly, we validated our selected models, including the “most-representative” model.

### Step 1. Produce candidate models

We used nonparametric cluster analysis to generate “candidate models” containing potential weather associations. Nonparametric cluster analysis can detect clusters of different shapes, sizes and dispersion, and thus provides more power for capturing potentially interesting patterns than more commonly used hierarchal techniques [76]. Analyses were conducted with the MODECLUS procedure of SAS 9.3 (SAS Institute Inc., Gary, NC), using method = 1 and a minimum cluster size of 50 (CK = 50). In the procedure, we altered the radius parameter (R) to produce analyses that varied in the kinds and numbers of clusters produced. About 200 candidate models were produced, each having between 2 to 8 clusters (we reasoned that models with more than 8 potential seasons were unlikely to be useful). Each “cluster number” was represented by at least 10 analyses. Weather variables were standardized before being entered into the analyses so that each had equal weight in influencing the results. Because nonparametric cluster analyses are sensitive to the influence of outliers, we examined normality of the frequency distribution of each variable using box-whisker plots. We found that the distribution of soil moisture was strongly positively skewed, so we normalized it using a natural log transformation. Similar approaches using cluster analyses have been employed to search for various associations among weather variables (e.g., synoptic types, weather regimes, or sub-seasons) [27, 77–80].

### Step 2. First culling of candidate models

We did not assume that all of the fire-weather variables would associate with each other in ways that allowed candidate models to be determined. It was possible that one or more of the variables would act as a “wrench in the works” and not allow useful models to be discovered [76]. We attempted to identify such variables and eliminate them from further consideration. We did this by testing data sets that contained different combinations of the fire-weather variables. See the Results and S1 Appendix (Section S1–1) for more details.

Our procedure generated hundreds of possible models. We did an initial culling based on the fit of models to fire-weather variables. We generated *R^2^* scores for each fire-weather variable of each model using an ANOVA (i.e., with cluster as the independent variable and the fire-weather variable as the independent variable). We then averaged the *R^2^* scores across the fire-weather variables within each model. The models were then plotted against each other in a scatter plot of mean *R^2^* score versus R, the cluster radius. This examination produced patterns that allowed models to be compared and evaluated. In addition to mean *R^2^* scores, we also tested other measures of model fit (AIC and Pillai’s trace). These other measures provided results almost identical to that provided by mean *R^2^* scores; we report mean *R^2^* scores here because they also provided intuitive information about models. Results for the other methods are detailed in S1 Appendix (Section S1–2). S1 Appendix (Section S1–3) contains an alternate method for culling models based on the number of days per year that were grouped into seasons. This method, especially combined with fit statistics, generated the same pattern for culling of candidate models.

### Step 3. Seasonality

Once a set of models was selected in Step 2, we then examined them to see if they contained seasonal patterns. For a given candidate model, clusters should contain both a reasonable number of days per year and unique seasonal timing to be considered as a season. Therefore, for each cluster, we examined its average number of days per year and how its days were distributed across the year. Models with one or more clusters that did not meet these criteria were eliminated from further consideration as potential fire-weather seasons. In this way, we eliminated short-term weather changes not consistent across years.

Once we had selected models with consistent seasons of at least 10 days across all years of the study, we ranked the models based on fit statistics. S1 Appendix (Section S1–4) contains the rankings of the fit statistics of these models. We used the mean of the rankings to select what we termed the most “representative model” for use in further analysis.

### Step 4. Examine fire-weather

We determined how effectively the “most-representative” model described fire-weather. We used discriminant analysis, a technique often used in SAS in conjunction with cluster analysis [27], (SAS Institute Inc., Gary, NC). Discriminant analysis decomposes multivariate data into composite variables (discriminant functions) that can predict which class a given observation belongs [81]. Thus, if a cluster analysis is successful, a discriminant analysis of the input variables of that cluster analysis should be able to predict the clusters to which the observations belong (i.e., with low percentages of miss-classified observations). Moreover, because each observation is given a score on each of the discriminant functions, discriminant plane graphs can be created that describe how clusters differ on the functions. We found that such visualizations effectively described relationships between fire weather and clusters of given candidate models and especially for the “most-representative” model. Discriminant analysis was performed using the DISCRIM procedure of SAS version 9.3. We specified the CANONICAL option (so that output included discriminant functions) as well as the POOL = NO option (to perform quadratic discriminant analysis).

### Step 5. Examination of fires in relation to fire-weather models

We also examined how well the “most-representative” model described fire regimes at the APAFR. Data on ignition dates, areas of fires, and source of ignition (lightning, military and prescribed) were obtained from a 13-year fire record (1997 to 2009). We mapped each fire recorded at the APAFR on the graphs produced by discriminant analysis. The size of these fires was detailed using bubble plots in which size of the bubble reflected area burned. Different plots were produced for the three ignition sources. We then examined how sizes and seasonal patterns resulting from fires with different ignition methods differed in their relationships to fire-weather and seasonal climate. We also described fire-weather conditions under which fires burned. This was done separately for fires stemming from the three ignitions sources and within different clusters of selected models. Average conditions were calculated for each fire type and cluster.

### Step 6. Validation of selected models

In addition to these five steps, we addressed the validity of selected models. First, we recognized that because our analyses attempt to reveal seasons (most importantly, whether there was a fire season), it was important to compare these analyses to a conventional description of seasonality at the site. Therefore, we compared our results to the prior description of seasonality based on rainfall [2], which used cumulative rainfall anomalies to define wet and dry seasons in the region. Second, we tested our key analyses to estimate how well they would perform if provided new data. For cluster analysis, we validated each selected model by randomly partitioning its data set into smaller data sets, and then repeating our analyses (Step 1 through 4 above) on each partition. Models were considered validated if they produced similar patterns and interpretations as the analysis of the full data set. Secondly, we examined the validity of discriminant analyses by examining their misclassification rate. We also performed a ‘leave-one-out’ cross-validation of these analyses. In the Results we discuss which specific models we ended up validating, and the outcomes of these procedures. Additional details on these analyses are provided in S1 Appendix (Section S1–5) and S1 Appendix (Section S1–6).

## Results

### Steps 1 and 2. Production of candidate models and initial culling

In our initial exploration, we performed cluster analyses on a data set that included all five fire-weather variables (relative humidity, solar radiation, soil moisture, air temperature, and wind speed). We found that, as expected, the overall fit of the models increased as more clusters were added (S1 Appendix, Section S1–1). However, none of the models stood out as being substantially more fit than the others, and thus we considered none of them as being “better” or potentially useful. We therefore proceeded to produce candidate models using data sets that included the possible combinations of four fire-weather variables. Examination of the fit of these models led us to conclude that none of the models that included wind speed stood out as having particularly good model fit (S1 Appendix, Section S1–1). Wind speed therefore appeared to associate with the other variables in such a way that prevented interesting candidate models from being produced, and so it was designated as a “wrench in the works” and eliminated from further consideration.

**Figure 1:**
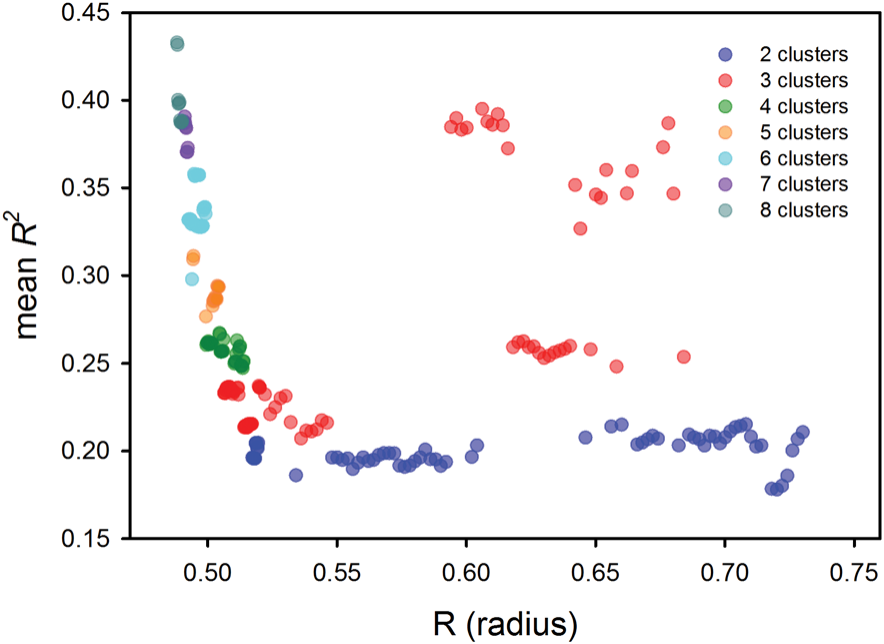
Model fit (mean *R^2^* scores) of candidate models developed to represent fire-weather seasons. Candidate models were generated by varying a parameter describing the radius used in the algorithm of the nonparametric cluster analyses. Models shown here were developed using four daily fire-weather variables (not wind speed) over 13 years (1997–2009).

The four variable data set containing relative humidity, solar radiation, soil moisture, and air temperature did produce viable models. We graphed mean *R^2^* scores against R, the cluster radius (Fig. 1). A set of 20 models with three clusters had mean *R^2^* scores ranging from 0.33 to 0.39. These scores were far higher than those of all of the models with 2 clusters, and were also substantially higher than other models with 3 clusters as well as all of the 4- to 5-cluster models. Moreover, these scores were comparable to those of the 6- and 7-cluster models, and were only surpassed by scores of some of the 8-cluster models. Considering that models with 6 or more clusters should be much more complex than models with 3 clusters, we considered them to lack parsimony and thus not to be as useful as 3 cluster models. Similar results were found for the “non-wind” data set when we examined other measures of model fit (Pillai’s trace, mean AIC scores) (S1 Appendix, Section S1–2).

In these comparisons among the candidate models, we also determined the mean *R^2^* score for our reference seasonal model (i.e., our model of wet and dry seasons derived using cumulative rainfall anomalies, CRAs). This model had a mean *R^2^* score of 0.134, which was lower than the *R^2^* scores of all models produced by cluster analysis of the four weather variables.

### Steps 3 and 4. Seasonality and fire-weather of the selected models

We considered the set of 3-cluster models with *R^2^* scores from 0.33 to 0.39 in the next phase of analysis. In Steps 3 and 4 we characterized the seasonality of the 20 selected models, as well as how effectively they described fire weather. Not only did all of the 20 models have clusters that appeared to represent a season, the picture they described of seasonality was similar. This result is not unexpected given the similarity of their R values (roughly 0.6–0.7; Fig. 1). We detail these cluster analyses in S1 Appendix (Section S1–4). Here, we present results from a representative model that appeared to be the “best” in terms of several model-fit criteria (see S1 Appendix, Section S1–4 for details). Although this representative model is useful, we want to emphasize that the most important finding here is that our analysis reveals a region in the “hyperspace” of four predictor variables where 3-cluster models successfully defined a third season.

**Figure 2:**
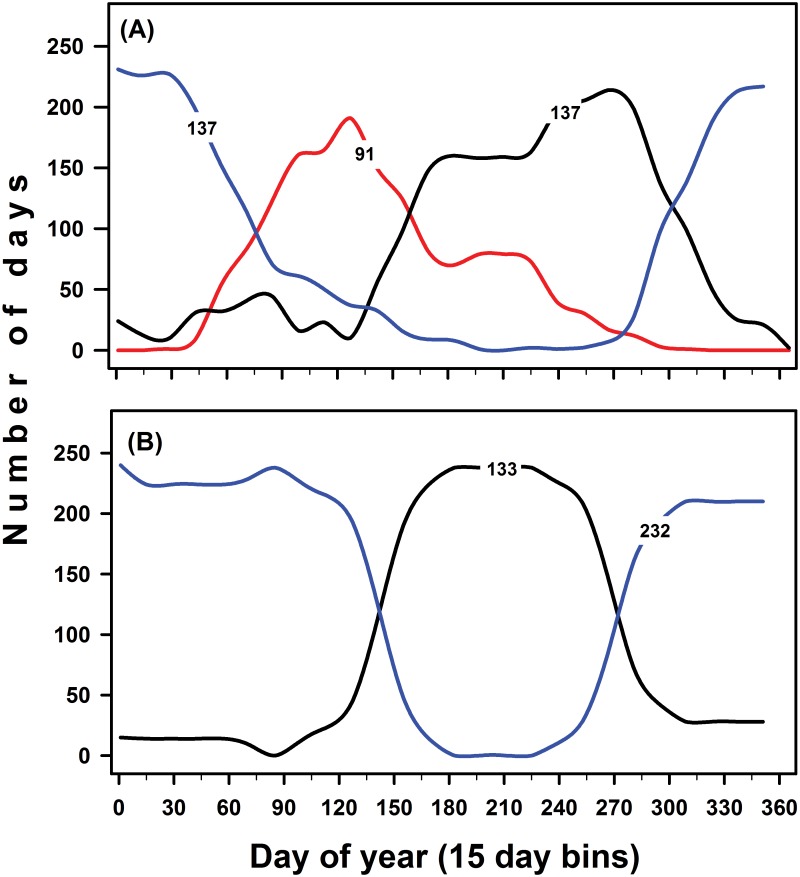
Seasonal timing of candidate models representing seasons. Models were developed using cluster analyses of four daily fire-weather variables over 13 years (1997–2009). Panels include (A) results of the selected model with 3 clusters (the “representative model”), and (B) results based on dry and wet seasons defined by the CRA reference model. For each panel histograms for different colors are coded as follows: black = histogram of cluster appearing to represent the wet season, blue = cluster appearing to represent the dry season, and red = cluster appearing to represent the peak fire season. Histograms use 15 day bins. Numbers detail average number of days per year found for each cluster.

The seasonal profile of this representative model is shown in Fig. 2. Cluster #1 was the predominant cluster for 137 days per year (days 156 to 323), approximately June to mid-November (Fig. 2A). This cluster loosely corresponded to the wet season, with similar days (133) and timing to the conventional model of seasonality based on rainfall (Fig. 2B). Cluster #2 was the predominant cluster from mid-October (day of year 295) to early April (day of year 99), also 137 days per year (Fig. 2A). This cluster loosely corresponded to the dry season in the conventional model based on rainfall (Fig. 2B). Cluster #3 was the predominant season from early April to late June (days of year 98 to 173), 91 days per year (Fig. 2A). The seasonal timing was coincident with the latter part of the dry season and the initial part of the wet season by the rainfall model (compare Fig. 2A and B). In addition, there was a “tail” of days that extended into the middle of the wet season defined using either model.

Discriminant analysis of the representative model showed that these three seasons had clear differences in fire weather. The analysis produced two discriminant functions, with the first explaining 64% of the variation and the second 36% (Table 1). These functions were both highly statistically significant (*F* values > 1000, *P* values < 0.0001). The first function described a trend of increasing solar radiation and temperature, while the second described a trend of increasing soil moisture and relative humidity, as well as a moderate decreasing trend in solar radiation (Table 1).

**Table 1:** Results of discriminant analysis showing the relationships between two discriminant functions and four variables describing fire weather conditions over 13 years (1997–2009) at the Avon Park Air Force Range, south-central Florida (USA).

**Discriminant function**	**Fire-weather variables**	**Structural correlations**	**Variation explained (%)**
I	Soil moisture	0.29	64%
	Relative humidity	-0.20	
	Solar radiation	0.87	
	Air temperature	0.94	
II	Soil moisture	0.96	36%
	Relative humidity	0.98	
	Solar radiation	-0.49	
	Air temperature	0.34	

**Figure 3:**
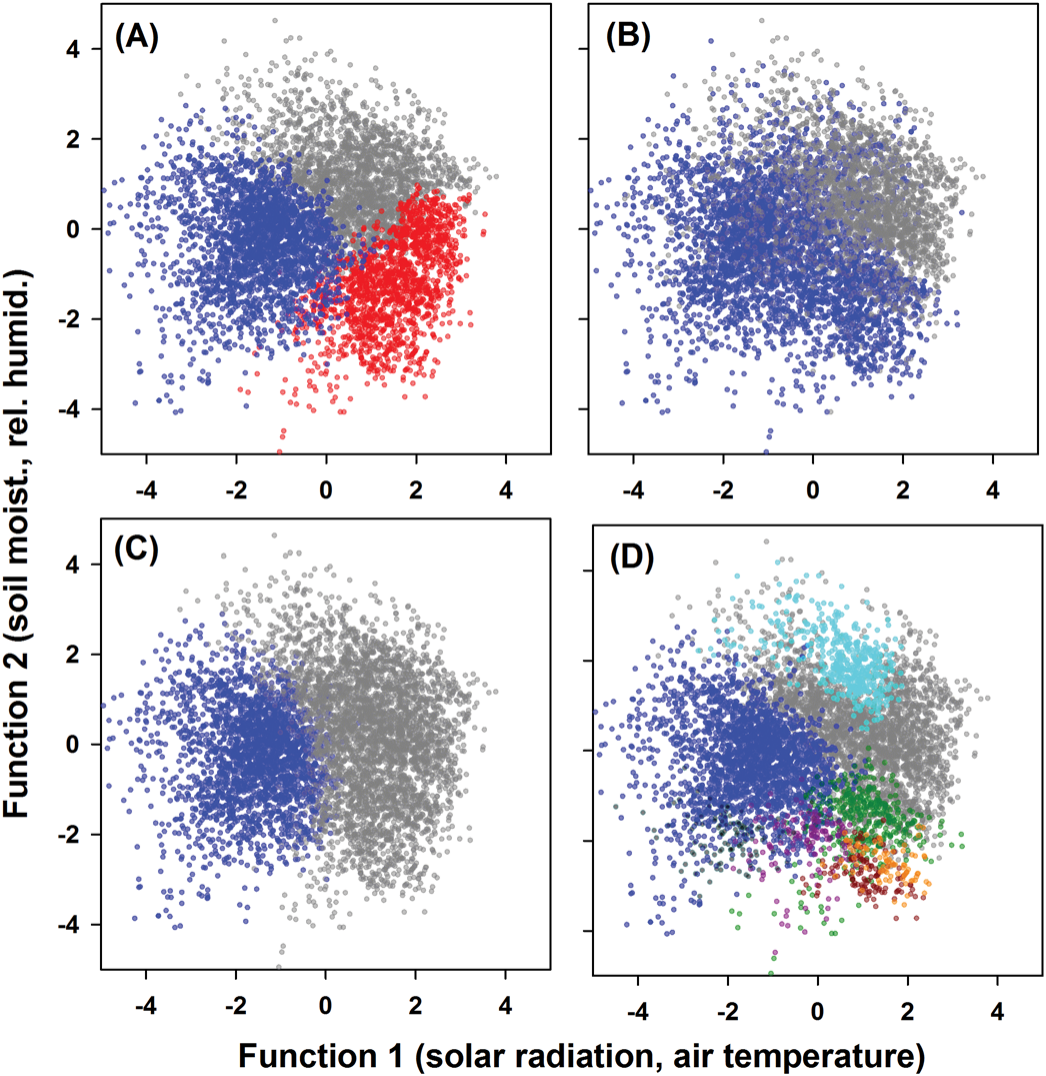
The “fire-weather plane” describing 13 years of fire weather. The plane is defined by the two discriminant functions described in Table 1. Panels include: (A) the seasons of the representative model (wet season, gray dots; dry season, blue dots; fire season, red dots). (B) The wet and dry seasons of the CRA model. (C) The wet and dry season of the “best” model with 2 clusters. (D) The “best” model with 8 clusters (clusters 3–8 are colored differently than the seasons found in the other panels).

We used a discriminant plane that we termed a “fire-weather plane” to explore relationships between fire weather and the days assigned to different clusters. We plotted the days assigned to the different clusters (seasons) in two dimensions (Fig. 3A). On this plane, Cluster #1 (the wet season) was located in the upper region of the plane, a placement that corresponded with its high soil moisture and relative humidity (Table 2). Soil moistures typically were low early in the wet season, but increased and often resulted in flooded conditions late in the wet season (82); these patterns of change resulted in much higher variation among wet season soil moistures than the other seasons, in which soil moisture tended to be much lower and more uniform (Table 2). In addition, more of the days also had warm air temperatures and high relative humidity (Table 2), resulting in most days being located in the upper right hand region of the fire-weather plane. Cluster # 2 (the dry season) was placed in the mid- and lower left region of the plane. Lower air temperatures and solar radiation, coupled with lower soil moisture and relative humidity, resulted in most days in the dry season being in the lower left region of the fire plane (Table 2). During the dry season, soil moistures declined rapidly from higher values at the end of the wet season to low values during the middle and end of the dry season. Cluster #3 was placed in the lower right hand region. Warmer air temperatures than the dry season and more intense solar radiation resulted from more clear days than in either the wet or dry season. These patterns were coupled with lower soil moisture and relative humidity than in either the wet or dry season and resulted in a distinctive cluster (Table 2). Because these conditions were favorable for fire spread, we labeled this third cluster the fire season. The two conventional seasons had less favorable conditions for fire spread, so the fire season was distinct as a separate season.

**Table 2:** Descriptions of fire-weather conditions as delimited by the representative 3-season model.

**Season**	**Wet**	**Dry**	**Fire**
Air temperature (°C)	24.9 ± 2.4	18.1 ± 4.1	24.2 ± 3.7
Relative humidity (%)	83 ± 5	78 ± 8	72 ± 6
Solar radiation (mW m^-2^)	0.19 ± 0.05	0.15 ± 0.05	0.28 ± 0.03
Soil moisture @ 30–60 cm depth (mm)	50.0 ± 34.2	21.5 ± 16.3	19.2 ± 11.5
Seasonal timing (day of year)	225 ± 71 (August 13)	20 ± 54 (January 20)	149 ± 53 (May 29)

All values are mean ± 1 standard deviation.

We gained further insight into the fire-weather at the APAFR by using the fire-weather plane of the representative model to examine some of the other models. We started with the CRA model (Fig. 3B) and the 2-cluster model with the highest mean *R^2^* score (Fig. 3C). When compared to the representative 3-season model, the CRA model split the days assigned to the fire season between the dry and wet seasons (compare Fig. 3B to 3A). In contrast, the “best” 2-cluster model assigned most of the days of the fire season in the representative 3-season model to the wet season (compare Fig. 3A and C). These results demonstrate the low explanatory power of 2-season models, as many days with unique fire weather characteristics are forced to belong to one of two clusters.

Models with more than three clusters tended to subdivide that region of the fire weather plane that contained the fire season in the 3-season model. For example, the 8-cluster model (Fig. 3D) produced four clusters within the cluster that defined the fire season, carved off that part of the dry season with intermediate values for function 1 and low values for function 2, and also separated one cluster within the wet season (compare Fig. 3A and D). This model also assigned part of the fire season to the wet season. Overall, results suggested that the fire season—especially that portion of the transition season with low values of function 2 (i.e., low relative humidity and soil moisture)—had more variable fire weather than the other seasonal modes.

### Step 5. Examination of fires

We examined relationships between characteristics of fires at APAFR with different sources of ignition (lightning, military, prescribed) and the three seasons of the representative model. Numbers of fires and area burned differed among the three ignition sources (Table 3). Most lightning-ignited fires occurred during the fire season (73%), with smaller proportions occurring during the wet (22%) and dry (5%) seasons. By far the most of the area burned occurred in the fire season (90%), with 8% and 2% of the area burned occurring, respectively, in the dry and wet seasons. Military fires occurred in both dry (39%) and fire (46%) seasons, but the area burned during the fire season (61%) was about twice that burned in the dry season (32%). Only 15% of military fires occurred in the wet season, and they only burned 6% of the total area burned in military fires. Over half (51%) of the prescribed fires occurred during the dry season; 31% and 18% occurred within the fire and wet seasons, respectively. These numbers were reflected in the areas burned (55, 33, and 12%, respectively, in dry, fire, and wet seasons).

**Table 3:** Descriptions of lightning, military, and prescribed fire regimes at the Avon Park Air Force Range, south-central Florida, USA (1997–2009).

**Season**	**Wet**	**Dry**	**Fire**
**Lightning Fires**			
Number of fires	13	3	43
Area burned (ha)	152	521	6,093
Air temperature (°C)	26.2 ± 0.8	23.4 ± 0.1	26.5 ± 3.0
Relative humidity (%)	85 ± 4	79 ± 1	74 ± 5
Solar radiation (mW m^-2^)	0.21 ± 0.04	0.20 ± 0.01	0.28 ± 0.02
Soil moisture @ 30–60 cm depth (mm)	32 ± 17	7 ± 4	14 ± 9
Seasonal timing (day of year)	210 ± 29 (Jul 29)	109 ± 42 (Apr 19)	163 ± 23 (Jun 12)
**Military fires**			
Number of fires	17	44	51
Area burned (ha)	1,154	5,853	11,115
Air temperature (°C)	25.6 ± 1.7	17.5 ± 4.3	23.3 ± 3.6
Relative humidity (%)	79 ± 5	75 ± 10	68 ± 6
Solar radiation (mW m^-2^)	0.22 ± 0.02	0.17 ± 0.05	0.28 ± 0.03
Soil moisture @ 30–60 cm depth (mm)	32 ± 27	17 ± 11	10 ± 6
Seasonal timing (day of year)	191 ± 52 (Jul 10)	36 ± 42 (Feb 5)	121 ± 31 (May 1)
**Prescribed fires**			
Number of fires	118	341	205
Area burned (ha)	15,269	69,681	41,129
Air temperature (°C)	23.9 ± 3.0	17.0 ± 4.4	23.4 ± 4.0
Relative humidity (%)	81 ± 5	75 ± 8	71 ± 6
Solar radiation (mW m^-2^)	0.21 ± 0.04	0.17 ± 0.04	0.28 ± 0.03
Soil moisture @ 30–60 cm depth (mm)	50 ± 35	23 ± 20	18 ± 9
Seasonal timing (day of year)	157 ± 81 (Jun 6)	40 ± 38 (Feb 9)	

For fires, data are sums over the period of record. For weather conditions, data are means ± 1 standard deviation. Data are grouped by the three seasons as produced by the representative 3-season model. Values for dry season are adjusted to include days spanning across different years.

Fire weather variables tended to be more associated with seasons than ignition sources. Large variation occurred in individual fire weather variables at the time of ignition, regardless of source (Table 3), suggesting that combinations of variables were more important than single variables in whether a fire occurred on a particular day. The most striking association of fire weather variables with ignition sources was that lightning and military fires tended to burn under lower soil moistures than prescribed fires in the same season (Table 3). These may have reflected, in part, seasonal timing differences, as the lightning fires in all seasons occurred, on average, later in the year than military or prescribed fires in these seasons.

**Figure 4:**
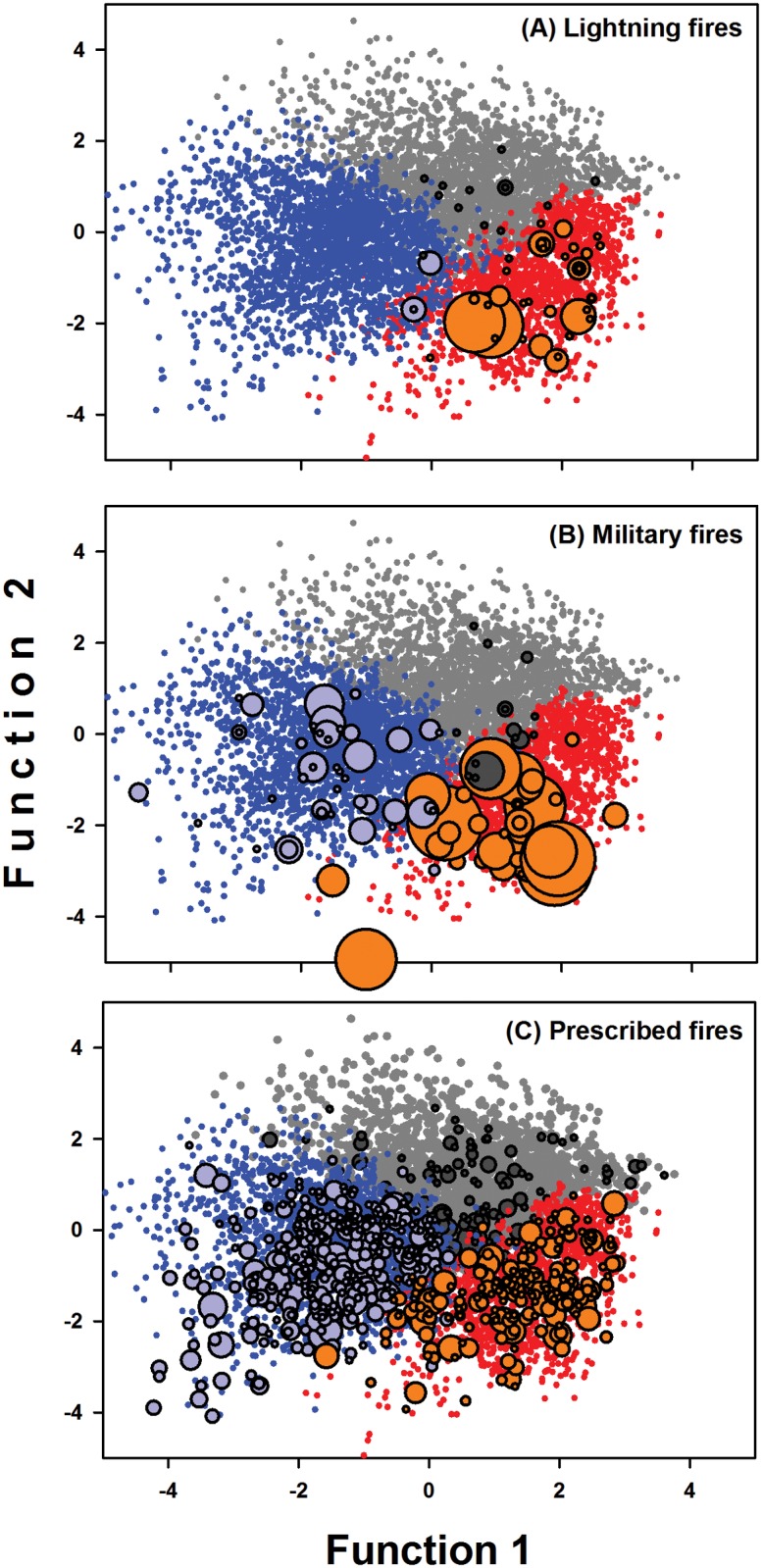
Relationships between the size of fires and climate as described on the fire-weather plane. Fire size is shown with bubble plots, with the size of bubbles being proportional to the size of fires. Shown are (A) lightning fires (n = 59), (B) military fires (n = 112), and (C) prescribed burns (n = 664). The seasons of the selected model are shown with different colored dots (wet season, gray dots; dry season, blue dots; peak fire season, red dots). Bubbles detailing fire size are filled with colors related to the season in which they occur (wet season fires, dark gray; dry season fires, light blue; peak fire season fires, orange). To make the relationships for prescribed burns clearer, we reduced the bubble size in panel C by 50% relative to that found in panels A and B.

We used the fire-weather plane to explore variation in climate conditions associated with fires of a given ignition source. We plotted lightning, military and prescribed fires separately on the fire-weather plane (Fig. 4). Noticeable patterns among seasons and fire types occurred for the largest wildfires. During the period of study, the APAFR had five wildfires >1000 ha, three military and two lightning fires; together these five fires accounted for 30% of the total area burned by wildfires. On the fire-weather plane, all of these fires occurred in the same region of the fire season area of the fire-weather plane (Fig. 4A, B). Almost all other sizable lightning- and military-ignited fires also occurred in this same region of the fire weather plane, suggesting that large fires occurred under restricted fire-weather conditions. This region was characterized by high solar radiation and air temperatures, and by low relative humidities and soil moistures. Prescribed fires, on the other hand, appeared to have been conducted to avoid this “peak fire season” of the plane; there were few prescribed fires conducted under fire weather conditions that resulted in large lightning and military fires (Fig. 4C). We further noticed that this part of the fire-weather plane was also the area that tended to have additional clusters when models with larger numbers of clusters were examined (compare Fig. 4 to Fig. 3D), suggesting that large fires may be associated with specific conditions within the fire season.

We viewed this last point as not being coincidental—there was likely something about fire weather in this region of the fire-weather plane that resulted in large wildfires. We therefore examined how wildfires varied among the different clusters of the 8-cluster model with the highest average R^2^ value. Half of the 10 largest lightning fires were concentrated within a single cluster (cluster #7; Table 4A). This cluster, which accounted for 69% of the total area burned by lightning fires (4,668 out of 6,766 ha total), is depicted in the lower region of the fire season in Fig. 4A. These fires burned under favorable conditions: warm air temperatures, high levels of solar radiation, low soil moisture, and moderate levels of humidity (Table 4A). The seasonal timing of these fires was tightly clustered around from the end of May to the end of June. Based on this analysis, it appeared that cluster #7 constituted the “peak mode” for lightning fires.

**Table 4:** Top 10 largest lightning and military fires, and the weather conditions under which they burned.

**Rank**	**Cluster**	**Size (ha)**	**Date**	**Air temperature (°C)**	**Relative humidity (%)**	**Solar radiation (mW m^-2^)**	**Soil moisture @ 30–60 cm (mm)**
**Lightning Fires**							
1*	7	1,914	5/27/2006	25	77	0.26	4.9
2*	7	1,652	6/24/2000	25	82	0.25	3.7
3*	7	549	6/22/1998	28	75	0.30	7.1
4	1	297	6/12/2007	26	73	0.26	27.5
5	3	269	5/13/2009	23	80	0.21	4.5
6	3	252	3/2/1997	23	77	0.20	12.0
7	7	247	5/28/2006	27	69	0.29	4.8
8*	7	241	6/6/2000	28	71	0.30	3.2
9	1	226	6/17/2001	26	79	0.31	19.4
10	4	181	5/7/1997	22	70	0.28	18.6
**Military fires**							
1*	6	2,128	5/4/2006	24	61	0.32	7.2
2*	4	1,405	3/7/2001	13	48	0.29	3.5
3*	7	1,038	6/15/2000	27	72	0.29	3.4
4*	6	748	5/4/2006	24	61	0.32	7.2
5	3	674	2/12/1999	21	86	0.12	19.7
6	1	651	6/8/1998	26	82	0.23	9.3
7	3	557	12/15/2007	23	86	0.10	9.5
8	5	547	3/31/2006	20	70	0.28	13.3
9*	6	506	5/4/2006	24	61	0.32	7.2
10	3	452	1/7/1997	21	79	0.16	11.8

Rows with rank followed by * indicate clusters constituting peak mode of the fire season.

Military ignition of fires occurred under a wider range of conditions than lightning ignition of fires. The ten largest fires burned under six of the eight clusters in the 8-cluster model (Table 4B); only the wet season had no large military fires. The most important cluster was #6, with three of the top 10 largest military wildfires that altogether accounted for 30% of the total area burned by military ignitions (5,490 ha out of 18,122 ha total). These three fires all occurred on May 4^th^, 2006, which had almost ideal conditions for fire. The 3^rd^ largest military fire occurred during the “peak mode” cluster of the lightning fire regime (#7), and had similar seasonal timing and weather conditions as those fires. On the fire-weather plane, cluster #7 was placed to adjacent to cluster #6, so these clusters occurred under very similar fire-weather conditions, resulting in large fires in the lower region of the fire season on the fire weather plane (Fig. 4B). The 2^nd^ largest military fire occurred in cluster #4. This fire occurred under fire-weather conditions that differed from other large wildfires; it burned on March 7^th^, 2001 under cool air temperatures, intense solar radiation, low soil moisture, and very low relative humidity (for Florida). The unusual character of this fire is readily shown on the fire-weather plane (it was placed to the left and below other large fires in Fig. 4B). Altogether, it appeared that, compared to the lightning fire peak mode, the military fires were spread over a range of possible weather conditions, but with a strong tendency to burn largest areas during the fire season under conditions similar to those under which largest lightning-ignited fires occurred.

We examined prescribed burns that occurred within those clusters where the largest wildfires occurred. Little prescribed burning was conducted in clusters 4, 6 and 7. Less than 2% of the total area burned by prescribed fires occurred on days within each of clusters 6 and 7. In contrast, considerably more burning was conducted within cluster 4 (10% of the total).

### Step 6. Validation of selected models

Our tests on the validity of our analyses indicated that they were valid and should be stable if more data were to be incorporated. First, we examined our representative cluster-analysis (S1 Appendix, Section S1–5). This examination revealed that a random partitioning of the data into four parts, and subsequent analysis, produced fire-weather planes very similar to those of the full data set (compare Fig. 3A to the figure that follows Section S1–5 in S1 Appendix). Similarly, we checked the results of the discriminant analysis of the representative model (S1 Appendix, Section S1–6). This examination revealed that the misclassification rate was low, around 10% (tables in S1 Appendix). Moreover, the observations that were erroneously classified were only found along the borders of the seasons, a result that is to be expected given that some days have to be transitional in nature (figures in S1 Appendix). Finally, a ‘leave-one-out’ cross-validation of this discriminant analysis produced a misclassification rate almost identical to that of the original analysis (S1 Appendix, Section S1–6). Given the results of these checks on the validity of our analyses, we concluded that, if we collected more data, it would be unlikely that analysis would produce seasonal models different from those already described.

## Discussion

Our concept of a fire season is rooted in prior studies of fire in southern Florida. Scientific notions of fire have changed considerably since fire in the south Florida region was initially mentioned by naturalists a century ago [83, 84]. Clear demonstration of the importance of fire in south Florida ecosystems dates to mid 20^th^ century studies by Robertson [85–87]. Over subsequent decades, seasonal timing and characteristics of lightning- and human-ignited fires have been described, and some of their effects on vegetation explored [32, 88–94]. Quantitative fire weather data have been used to predict aspects of lightning- and human-ignited fires in southern Florida, however, for only about a decade [2, 3, 20, 33, 44, 51, 65, 82]. These studies have considered the greatest likelihoods of fire and areas burned naturally by fire as resulting from lightning strikes during thunderstorms that end the dry season and initiate the ensuing wet season [33, 90, 95, 96]. Our current study builds on these prior studies, developing new concepts regarding the importance of seasonal timing in fire regimes of southern Florida savanna-grassland landscapes. We redefine seasons using fire weather, and the most useful seasonal model contains both wet and dry seasons, as well as a distinct fire season.

### A three-season model of fire weather for southern Florida

Seasonal components of fire regimes most often have been delineated based on single weather variables, such as temperature or rainfall. In our study, we developed multivariate models to demarcate seasons using combinations of weather variables that influence spread of fires across landscapes. Based on evaluation of model parsimony, the occurrence of model-designated seasons as a number of days each year, and unique seasonal timing, we selected a three-season model that explained almost twice as much variation in fire-weather variables in southern Florida as the conventional wet/dry season model based on rainfall [2, 33, 55]. These three seasons included wet and dry seasons resembling seasons delineated using conventional rainfall models, as well as a distinct fire season characterized by high solar radiation, warm ambient temperatures, moderate relative humidity, and low soil moisture. This combination of weather conditions facilitates the likelihood and spread of fire in savanna-grassland habitats. This fire season has unique seasonal timing and occurs as a continuous period each year that follows the dry season and is followed by the wet season. The fire season averages a little less than three months, and does not represent variable or transient weather associations (e.g., storm fronts). Weather delineating the fire season is distinct from that during either conventional dry and wet seasons because fire-weather variables are strongly linked during the fire season, producing unique weather not described by conventional rainfall models.

Our three-season model clarifies seasonal changes in fire-weather. Fire weather remains relatively consistent over much of the wet and dry seasons in south Florida. As the dry season transitions to the fire season, however, concurrent changes in solar radiation, air temperatures, and relative humidity result in drying of fuels and soils. What has been perceived as the end of the dry season [2, 61, 65], is now designated the onset of the fire season. The fire season, with fire weather conducive for ignition and fire spread, thus is in place well before the first thunderstorms. This shift from the dry season to the fire season is indicated in Fig. 2.

Concurrent changes in components of fire weather that define the fire season do not appear to be tightly synchronized. As a result, variable weather conditions often occur during the fire season. Some years, for instance, appear to have “false starts” to the wet season, when a rainy period interrupts the fire season [2]. These often seem to be a harbinger of the beginning of the wet season, but the wet season fails to develop, and the “false start” is followed by another period of drought [2, 56]. Such variation may be conducive to fires, with multiple thunderstorms occurring under conditions favorable for fire spread before the wet season. Thus, conditions previously designated as the onset of the wet season [2, 61, 65, 82], based on rainfall now constitute the end of the fire season. The three-season model removes almost all natural lightning-ignited fires, and especially those that burn large areas, from the wet season. As a result, only small amounts of area are burned during the wet season, even though there are frequent lightning strikes that start fires during this season. Depending on how quickly soil catena become saturated and moisture conditions become less suitable for fire spread, the fire season can extend into what has been perceived as the early wet season [61, 65, 82]. We further anticipate that variation in assignment of days to fire and wet seasons is likely to result from global weather patterns such as ENSO [2, 3, 33]. Further study should refine concepts regarding intra- and inter-annual variability in weather conditions during the fire season and how such variation contributes to pyrodiversity.

Models with more than three seasons provide additional insights into the fire season of the selected three-season model. Additional clusters of fire-weather variables produced by rejected models with >3 clusters tend to be grouped within the fire season of the three-season model. These patterns suggest that the fire season in south Florida is characterized by more variable fire weather conditions than either the dry or wet seasons. In addition, some of these subdivisions of the fire season are strongly associated with large lightning and military fires, suggesting that conditions conducive for fire spread across landscapes (peak fire season) occur under highly specific fire-weather conditions that are somewhat repetitive across years.

Fires during peak fire season may be crucial for scientific-based restoration and management of certain habitats. The peak fire season involves weather conditions sufficiently dry for fires to cross wetlands that otherwise tend to serve as natural firebreaks (at the end of the fire season, during the entire wet season, and into most of the dry season). Thus, prescribed fire in south Florida often has been tantamount to fire suppression in low-lying or transition areas between wetlands and uplands. One result has been transformation of herbaceous-dominated wetland transitions to shrub-encroached thickets (i.e., fire breaks that burn only in intense fires during extreme droughts) at the expense of a diverse groundcover often rich with endemic plants [82, 97–99]. These patterns suggest that scientifically-based fire management of longer-hydroperiod wetlands could be enhanced by exploration of characteristics of peak fire seasons. Understanding ecological effects resulting from human ignitions that vary from natural fire regimes should provide a scientific basis for modifying ignitions in ways that benefit natural resource management priorities such as ecosystem management, conservation of biodiversity, and maintaining endangered species habitat.

We suggest that the most appropriate fire-weather models are likely to be local, or at most regional in scale. Those fire-weather variables in our models are similar, but not the same as weather variables that tend to be used in more general fire models. For example, fire-spread models such as BEHAVE and its derivatives have been broadly applied across geographic regions [100–103]; see (http://www.wfas.net/). These models may not incorporate the most appropriate fire weather variables (and also may contain too many such variables) for accurate description of a seasonal fire weather patterns at a given site. We propose that fire season models using variables known to affect fire directly at local scales should have a greater chance of identifying weather-fire relationships with high precision. In central and southern Florida, for example, our models using local weather variables that influence ignition and spread of fire over relatively short time periods are more accurate than more general models based on derived variables, such as fuel moisture or prolonged drought indices (e.g., Palmer or Keetch-Byram drought indices) that indicate longer-term deviation from saturated soils [2].

Regional or local models of fire seasons may not include all weather variables known to influence fires. Inclusion of variables that influence fire-weather, but not in a distinct intra-annual pattern, may not produce useful models of fire seasons. Therefore including such variables in the model will interfere with the ability of the model to detect seasonal clusters of days that change similarly over time. In our case with wind speed, one possible explanation is that windy days in southern Florida may tend to be days with stormy weather, and such weather occurs across seasons. For example, storm fronts occur periodically during the dry season, with the weather pushed before these fronts tending to be blustery, warm, and humid [104], resulting in transient weather are considerably different from fire weather patterns that more generally characterize the dry season. Thus, wind speed should not necessarily be considered aseasonal at APAFR, but its seasonality appears to operate differently than other fire-weather variables [51]. In other regions, however, wind (or maybe some subset of variables associated with wind) may be critical for defining fire seasons (e.g., foehn-type wind seasons such as the Santa Ana winds; [37]. Multivariate modeling of fire-weather seasons requires mechanisms for identifying the most suitable set for a site and removing other variables from consideration.

Given that our fire-weather analysis reveals a fire season between the dry season and subsequent wet season, a similar season might be expected between wet and dry seasons. The absence of a fourth season in southern Florida could result from lag effects of wet season precipitation that sustain water tables at or even above the ground surface for extended periods in low-lying areas [20, 82]. High soil moisture content (i.e. saturated, water-logged or inundated soils) thus would influence aspects of the fire weather conditions (e.g., drought conditions, relative humidity) after the wet season ends. Such a pattern is well-documented for the Everglades, where slow drainage of low-lying landscapes commonly extends into the dry season [33, 105], buffering the early dry season against favorable fire conditions [2, 106]. We note that in southeastern regions not low-lying and slow to drain, weather patterns could result in a four-season model that potentially includes two peak fire seasons, as suggested by relationships between rainfall and fire regimes further north along the Florida peninsula and Gulf coast [70, 107–109]. Even more different relationships might be discovered around the world.

### Relationships between fire weather, ignition sources, and fire regimes

Fire regimes in south Florida are dominated by landscape-level wildfires. Such fires overwhelmingly determine the frequency with which a given location burns [44, 51]. Although ignitions potentially can occur during the entire year, fires burn the vast majority of area only in the fire season. Our analyses further suggest that lightning and military fire regimes at the APAFR differ to the extent that variation in seasonal timing of ignition results from differences in fire weather at highly specific times—peak fire mode—during the fire season. Both lightning and military fire regimes, as well as area burned by each ignition type, are constrained by seasonal timing of ignition relative to fire weather during the fire season.

Relationships between fire weather and fire spread are complex. Wildfires have been postulated to spread via a process of stepwise increases (stages) in size [110]. Our prior analyses [51] indicate that increases in size at smaller stages at the APAFR are “promoted” by low relative humidity and higher wind speeds. Only as fire size increases to larger stages do the full effects of drought become realized, because lower elevations become suitable fire-connections between upland areas at these larger sizes [51]. Although the stepwise process allows fires to become large (> 1000 ha for the APAFR), it does not mandate that they will become large fires. Our current analysis reveals that this stepwise process results in large fire size in southern Florida only under critical peak fire weather conditions during the fire season.

Our analyses indicate critical combinations of seasonal fire weather conditions govern fire spread. More conventional models, especially those based on indices of long-term drought conditions, e.g., [111, 112] and short-term fire danger ratings (e.g., [113, 114]; Wildland Fire Assessment System at http://www.wfas.net/) also indicate current and short-term future likelihoods of spread of wildfires. These models do little, however, to describe how important weather variables (and associated effects on fuels) come together to produce fire seasons, as well as identify peak fire modes within fire seasons. Our approach should be useful in predicting seasonality of large fires, an important management question in landscapes where “blow-ups” are possible because there are few effective natural barriers to the spread of fires. Eventually, our multivariate analytical approach should be combined with predictive models that incorporate intra- and inter-annual variation in multiple important fire weather variables [20].

Our three-season model reveals variation in fire regimes produced by different ignition sources at the APAFR in south-central Florida. The vast majority of area burned during natural lightning-ignited fires occurs during the peak fire mode of the fire season. Hence, the natural fire season is highly predictable, with important ecological consequences [82]. Fires are expected to be frequent and predictable in occurrence because peak fire mode occurs annually. Human ignitions, however, often pre-empt lightning ignitions [22]. Most area burned as a result of military missions occurs during peak fire mode or slightly earlier under similar fire conditions. Military missions tend to alter natural fire regimes only slightly, although, as we have noted, there are some exceptions. Prescribed fires result in the most noticeable shifts in fire regimes. In contrast to lightning and military fires, prescribed fires at the APAFR burn the most area and have the largest fires outside peak fire mode and even outside the fire season. Prescribed fires often are banned or not conducted during peak mode because they are considered more likely to escape, becoming hard-to-control wildfires. Thus, prescribed fires commonly are shifted earlier, into the end of the dry season, or later, into the end of the fire season or into the wet season. Other studies in Florida also have found that prescribed fires are rarely conducted during peak mode [44, 61]. Even planned experimental fires may be re-scheduled because of burn bans associated with peak mode [45]. Because prescribed fires often occur weeks-months prior to, or after lightning-ignited fires, under ecological conditions outside those in the natural fire season, considerable confusion exists concerning similarities and differences in ecological effects of fires during peak and non-peak modes, as well as inside and outside the fire season.

Fire regimes concentrated inside the fire season are likely to maintain savanna-grassland landscapes. Thus, fires that mimic natural fires (e.g., military missions) historically important in the evolution and ecology of frequently burned habitats like savannas and grasslands should not be detrimental and may facilitate fire management of fragmented landscapes. Decades of lightning and military fire regimes might have maintained savanna-grasslands within APAFR mission impact ranges especially at the low end of the moisture gradient, despite landscape fragmentation, see also [82]. Similar effects of human ignitions have been postulated elsewhere in the North American Coastal Plain [115].

How important is mimicry of natural lightning-ignited fires? Are there ecological effects of igniting fires during peak mode that do not occur otherwise? Does seasonal timing that fails to mimic the seasonality inherent in lightning-ignited fire regimes result in fires that might more appropriately be considered disturbances of natural fire regimes? It is common for decisions to be made that prescribed fires inside and outside the fire season produce similar effects based on lack of pronounced changes in upland habitat, despite phenological and demographic data from savannas and grasslands suggesting subtle effects [62, 99, 116–119]. We note, moreover, that in flatland landscapes of the southeastern coastal plain, prescribed fires during the mid to late wet season, when rises in water tables often can be measured in days and flooding follows soon afterwards, can be detrimental to groundcover vegetation. Large, often conspicuous negative effects occur when plants are top-killed, then flooded before resprouting can occur [33, 82, 93, 99, 120]. Such combined fire-flood effects occur frequently along lower ends of elevation gradients commonly measured in centimeters in south Florida [120–123]. Conversely a trend in southern Florida to shift prescribed fires into the late wet season or early dry season (September—December) fails to mimic fire season effects. Instead, fires are patchy and have reduced shrub top-kill, favoring re-sprouting woody species while failing to induce flowering of C4 grasses and reproduction of many groundcover forbs. We urge further study comparing effects of prescribed fires that mimic and do not mimic peak fire season timing on vegetation along moisture gradients in southern Florida.

### Coda: Implications for ecological fire management on a global scale

Our study provides a scientific basis for understanding differences between natural and human fire management of savannas and grasslands. Worldwide, humans have supplanted fire weather, suppressing or replacing fires during the peak mode of the fire season with fires of human design. Currently, production of fine fuels that ignite readily during droughts is at the periphery of planned restoration and management, and so human prescribed fires rarely simulate lightning fires in their seasonal timing across savanna-grassland landscapes. For many decades [6, 95, 115], prescribed fires in south Florida and similar habitats in other regions have been conducted over a wide range of seasonal times, almost the entire year [108]. Timing of ignition has been described using vague seasons defined using single weather variables (e.g., dormant (winter) versus growing (summer) seasons, or lightning versus non-lightning seasons). Hence, prescribed fires conducted during a wide range of times have been considered as having a “natural” seasonal timing; such fires often have been presumed (frequently without supporting data) to produce fire effects similar to those that would have occurred naturally under natural lightning fire regimes [124]. Consequently, confusion has resulted regarding similarities and differences in effects of fires at different times of the year.

Our study provides a compelling rationale for basing ecological fire management in analyses of relationships between fire weather and fires. The fire-weather variables we used are closely connected to local fuel conditions at the APAFR [2, 82, 99]. Perennial C_4_ grasses are dominant or co-dominant in savanna grasslands throughout peninsular Florida; at APAFR these grasses produce annual accumulations of fine fuel biomass across often subtle elevation gradients during the wet season [52]. Savanna pines (*Pinus palustris*, *Pinus elliottii* var. *densa*) and cypress (*Taxodium ascendens*) augment flammable fine fuels via leaf shedding [118]. Moreover, pyrogenic shrubs, especially palmetto (*Serenoa repens*) further modify fire characteristics [8]. Short-term, intra-annual droughts cure and connect these fine fuels across landscapes containing low, moisture-laden areas [34, 82]. Thus, lightning ignitions during peak fire mode of the fire season, when ground water levels are at their seasonal lows following rain-free intervals [2, 61, 82], have the potential to generate fires that readily burn these fine fuels, spreading over large areas annually, especially during La Nina induced droughts [2, 20, 33]. Such relatively specific seasonal timing, during peak mode of the fire season, sets the stage for evolutionarily-derived responses of dominant vegetation that affects likelihoods and characteristics of future fires [6]. Conservation of these habitats necessitates reconstitution of evolutionarily derived fire regimes.

Our conceptual fire model for south Florida provides the basis for a general evolutionary fire model for savannas and grasslands. In many landscapes worldwide, seasonal fire weather dries fine fuels, then provide the ignition source that results in fires that spread across landscapes, carried by highly flammable fuels that strongly influence fire characteristics. Our three-season fire weather model thus has conceptual implications for fire-vegetation relationships in humid seasonal savanna grasslands worldwide. Although seasonal weather patterns such as those in south Florida may appear specific, they also characterize humid warm temperate and subtropical habitats in many regions of the world. We anticipate fire weather models similar to that we develop for south Florida will have value in savannas and grasslands along the North American Coastal Plain [6, 121, 125–127], in the Caribbean basin, and in regions of Central and South America [95, 128–131]. Moreover, implications of a three-season model are likely to be important worldwide in temperate and tropical regions with savanna and grassland biomes [22, 132, 133]. These regions, especially those with a long evolutionary history, are likely to be overlooked, mismanaged, and under-appreciated as climate refugia of biodiversity and endemism important for conservation in a time of rapid climate change [134]. Hopefully, our analyses of fire weather, ignition patterns and fire regimes in south Florida will stimulate scientific evaluation of concepts underlying prescribed fire management worldwide.

## Supporting Information

S1 Appendix Procedures and technical details of data analyses. Section S1–1. Wind speed: a “wrench in the works” variable. Section S1–2. Fit statistics for selected models. Section S1–3. A second way to cull models: number of days per year. Section S1–4. Other “good” models with 3-clusters. Section S1–5. Stability of cluster analysis. Section S1–6. Discriminant analysis: percent misclassified and cross-validation.
(DOCX)
