# Supplementary material for: Seasonality of Fire Weather Strongly Influences Fire Regimes in South Florida Savanna-Grassland Landscapes
Source: PLoS One. 2015 Jan 9;10(1):e0116952. doi: 10.1371/journal.pone.0116952 (PMC4289074; doi:10.1371/journal.pone.0116952)
Supplement: S1 Appendix — Section S1–1. Wind speed: a “wrench in the works” variable. Section S1–2. Fit statistics for selected models. Section S1–3. A second way to cull models: number of days per year. Section S1–4. Other “good” models with 3-clusters. Section S1–5. Stability of cluster analysis. Section S1–6. Discriminant analysis: percent misclassified and cross-validation. (DOCX) [file pone.0116952.s001.docx]

**S1 Appendix**

We expand on our procedures and complement analyses in the main text with a set of accompanying analyses providing details. These include:

S1-1. Wind speed: a “wrench in the works” variable

S1-2. Fit statistics for selected models

S1-3. A second way to cull models: number of days per year

S1-4. Other “good” models with 3-clusters

S1-5. Stability of cluster analysis

S1-6. Discriminant analysis: percent misclassified and cross-validation

**S1-1. Wind speed: a “wrench in the works” variable**

Our exploration of data sets that included wind speed resulted in candidate models that tended to have rather low R^2^ (Figure S1-1). The patterns found for each of these data sets were different, but in no cases do we see a model or set of models that “separates from the pack” to the extent found with the twenty models we ended up selecting when using the “non-wind” data set. Our results reflect observations of Evervitt et al. (2011) [1], who state that the results of cluster analyses can change in important ways depending on which input variables are included. Some variables tend not to associate with the others in ways that are conducive for visualization of useful cluster patterns. For our study, it appeared that wind speed was such a variable.

**Figure S1-1.** **Mean *R^2^* scores of models produced by nonparametric cluster analysis using data sets that included wind speed.** Shown are results from five data sets. (A) Five variable fire weather data set (wind speed, air temperature, solar radiation, relative humidity, and soil moisture @ 30-60 cm depth). (B) The same variables as A, but not air temperature, (C) not soil moisture, (D) not solar radiation, and (E) not relative humidity. Symbols are given 50% transparency so that overlapping symbols can be more easily discerned.

[1] Everitt BS, Landau S, Leese M, Stahl D (2011) Cluster Analysis, 5th Edition. Chichester, West Susses, United Kingdom: Wiley.

**S1-2. Fit statistics for selected models**

Using the data set without wind speed, we identified twenty models with 3-clusters that appeared to have good fit in terms of mean *R^2^* scores. How well did these models perform when examining other fit statistics? Here we examined two other measures of fit, the Akaike Information Criterion (AIC) and Pillai’s trace. For the former, we took average values across the fire weather variables within a given model (i.e., just like we did with *R^2^* scores). For the latter, we ran a MANOVA on each model using cluster membership to predict the fire-weather variables. MANOVAs were run using the SAS GLM procedure. These examinations revealed results paralleling those found for mean *R^2^* scores, that is, each examination found a set of 20 models with 3-clusters that stood out from the rest of the models (Figure S1-2).

**Figure S1-2.** **Alternative measures of fit for candidate models produced for the “non-wind” data set.** (A) Akaike Information Criterion (AIC). (B) Pillai’s trace. Symbols are given 50% transparency so that overlapping symbols can be more easily discerned.

**S1-3. A second way to cull models: number of days per year**

We used fit statistics to cull our initial set of models. We noted, however, that we could have used number of days that were assigned to each cluster. Many, if not most, of the models had clusters that had very few days assigned to them per year (lower left in Figure S1-3). Also, many of the models had clusters that did not appear in one or more years of the study; this tendency for “zero day” models increased as cluster number increased. These models thus were not likely to represent annual seasons and were eliminated from further consideration. One set of 3-cluster models outside of our original 20 selected models did appear viable (shown with red circles in Figure S1-3). These models were eliminated based on model fit. We include this alternative method here because it reinforces the use of model fit to select candidate models.

**Figure S1-3.** **Number of days per cluster per year for the candidate models of the “non-wind” data set.** Number of clusters is color coded as per Figure S1-2. Squares indicate clusters of models that were selected based on mean *R^2^* scores in our main analysis; circles indicate clusters from models that were not selected. The clusters of the representative model are shown with crossed-squares. Models indicated with an “x” had at least one cluster that did not appear in all years of the study. Symbols are given 50% transparency so that overlapping symbols can be more easily discerned.

**S1-4. Other “good” models with 3-clusters**

Given that we had 20 models that were clearly a better fit than the other models, the next logical question arose: which of these 20 models provided the most useful description of fire-weather seasonality? To address this question, we ranked the models by their mean *R^2^*, mean AIC, and Pillai’s trace values (Table S1-1). Then, using this ranked list to give us perspective, we examined the seasonal profiles of the models as well as their fire-weather planes. This analysis revealed that, as model fit improved, the seasonal timing of the three seasons gradually became more defined, and a larger, more distinct third season was revealed on the fire-weather plane. To illustrate this, we detail results for the 2^nd^ “best” model (with a radius of 0.612) and the 20^th^ “best” model (radius of 0.644), comparing them to the “best” model described in the main text (i.e., the “representative” model). The results for the 2^nd^ “best” model were almost identical to those of the representative model (compare Figures S1-4A to Figure 2 and Figure S1-5A to Figure 3). The 20^th^ “best” model, on the other hand, clearly provided less useful patterns for understanding fire-weather seasonality, with a fire season that had a substantially reduced “signature” in the fire weather planes (Figures S1-4B, S1-5B). The other models examined (the 3^rd^ to 19^th^ “best”) had patterns intermediate between these two extremes.

**Figure S1-4.** **Seasonal timing profiles of two other models with three clusters.** These include: (A) the second “best” model (radius = 0.612) and (B) the 20^th^ “best” model (radius = 0.644). Lines show results for smoothed histograms using 15 day bins. The three clusters are colored coded as: black = histogram of the cluster representing the wet season, blue = the dry season, red = the fire season.

**Figure S1-5. Fire-weather planes of two other models with three clusters.** These include: the (A) second “best” model (radius = 0.612) and (B) the 20^th^ “best” model (radius = 0.644). Clusters are colored coded as: cluster representing the wet season = gray dots; dry season = blue dots; fire season = red dots. Symbols are given 50% transparency so that overlapping symbols can be more easily discerned.

**S1-5. Stability of cluster analysis**

The stability of an analysis can be defined as the ability of the analysis to be able to handle new data without these data changing the outcome of the analysis. Given that the set of cluster analyses of the non-wind data set produced the core of our results and conclusions, we conducted a measure of the stability of these analyses. We did this by randomly dividing the data set into four equal partitions, and by repeating Steps 1 to 4 of our investigation (see main text) on each partition. We found that:

1. For each partition, there was always a set of models with three clusters that appeared “good,” with the “best” of these models having a mean *R^2^* score of around 0.40.
2. Discriminant analyses of the “best” 3-cluster model of each partition resulted in functions with structural correlations very similar to those of the full model, that is, the first canonical function was heavily loaded by air temperature and solar radiation, while the second function was heavily loaded with humidity and soil moisture @ 30-60 cm depth (see Table 1).
3. Among the partitions, the fire-weather planes of the “best” 3-cluster models were very similar (Figure S1-6).
4. The seasonal profiles of these models had clusters with distinct seasonal timing and reasonable numbers of days per year (data not shown).
5. For some of the partitions there were models with four or five clusters that had high mean *R^2^* scores, but when we examined the seasonality of these models they either: (1) did not have reasonable numbers of days per year, with one or more clusters having < 30 days per year, or (2) did not have distinct seasonal timing.

Overall we concluded that our general technique for exploring for fire-weather seasons at our site appear to be stable. If new data were added to our analysis, it does not appear likely that they would produce an arrangement of seasons different from that already described.

**Figure S1-6. Fire-weather planes of four equal partitions of the “non-wind” data set.** Each plane was derived from a discriminant analysis of the candidate model with the highest mean *R^2^* score. Symbol are: wet season = gray dots; dry season = blue dots; fire season = red dots. Symbols are given 50% transparency so that overlapping symbols can be more easily discerned.

**S1-6. Discriminant analysis: percent misclassified and cross-validation**

Like with cluster analysis, investigators using discriminant analysis can examine their results to see if similar patterns would be found if more data were collected. Fortunately it is easy to examine the stability of a discriminant analysis. The way the success of a discriminant analysis is most commonly judged is by comparing predicted classifications versus real classifications, i.e., the “error rate”. For our representative 3-cluster model this error rate was low (around 9.4% overall) (Table S1-2). On the fire-weather plane, misclassified observations were generally placed along the borders of the seasons (Figure S1-7).

**(A) Number of observations and percent classified into the clusters**

**From Cluster**

**Fire**

**Wet**

**Dry**

**Total**

**Fire**

1,322 (85.1%)

152 (9.8%)

79 (5.1%)

1,553 (100%)

**Wet**

40 (1.7%)

2,134 (91.5%)

158 (6.8%)

2,332 (100%)

**Dry**

34 (1.5%)

119 (5.1%)

88 (93.5%)

2,341(100%)

**Total**

1,396 (22.4%)

2,405 (38.6%)

2,425 (38.6%)

6,226 (100%)

**Priors**

24.9%

37.5%

37.6%

**(B) Error count estimates for the clusters**

**Fire**

**Wet**

**Dry**

**Total**

**Rate**

14.9%

8.5%

6.5%

9.4%

**Priors**

24.9%

37.5%

37.6%

**Into Cluster**

**Cluster**

**Table S1**

**-**

**2.**

Error

rates of discriminant analysis in classifying observations

into their clusters (seasons).

**Figure S1-7. Fire-weather plane of the representative model with miss-classified observations.** Symbol are: wet season = gray dots; dry season = blue dots; fire season = red dots; black squares = observations that were classified as belonging to the wet season by the cluster analysis but miss-assigned by the discriminant analysis as belonging to the dry or fire seasons; teal squares = miss-assigned observations of the dry season; orange squares = miss-assigned observations of the fire season. Symbols are given 50% transparency so that overlapping symbols can be more easily discerned.

Thus, one of the main points is that, not only does the discriminant analysis assign the bulk of the observations to the correct seasons (ca. 90% success rate), those observations that are misclassified do not constitute a serious problem. In fact, we would expect that, when dealing with a multivariate data set detailing seasons, some days should likely be transitional and thus would be inherently difficult to assign to one season.

Like with cluster analysis, we were also interested in determining if new data would be accurately assigned to clusters when using discriminant analysis. To do this determination, we performed a k-fold cross-validation. The basic idea behind this technique is: (1) take, at random, *k* equal-sized partitions (or “folds”) of the data set, (2) set one fold aside as the “test” data set and combine the remaining folds into a “training” data set, (3) conduct discriminant analysis on the training data set to derive equations for predicting categories (seasons), (3) apply these equations to the test data set to derive a predicted category for each observation, (4) compare the predicted categorizations of the test data set to the training data in terms of percent misclassified, and finally, (5) repeat this process using different folds as the test and training data sets.

One commonly used type of cross-validation is called “leave-one-out” cross-validation [1]. In this case the data partition consists of a test data set of just one observation (the “left out” observation) and a training data set of the rest of the observations. This partitioning is repeated so that each observation is left out one time. The error rates are then averaged across all of the repeated analyses. In SAS, leave-one-out cross-validation is done using the using the CROSSVALIDATE option in the PROC DISCRIM statement. When we applied this to our twenty “good” 3-cluster models, we found that there was virtually no change in the percent misclassified when compared to the result without cross-validation (data not shown).
